# Enhanced Reduction of Ferredoxin in PGR5-Deficient Mutant of *Arabidopsis thaliana* Stimulated Ferredoxin-Dependent Cyclic Electron Flow around Photosystem I

**DOI:** 10.3390/ijms25052677

**Published:** 2024-02-26

**Authors:** Shu Maekawa, Miho Ohnishi, Shinya Wada, Kentaro Ifuku, Chikahiro Miyake

**Affiliations:** 1 Graduate School for Agricultural Science, Kobe University, 1-1 Rokkodai, Nada-Ku, Kobe 657-8501, Japanswada@penguin.kobe-u.ac.jp (S.W.); 2 Core Research for Evolutional Science and Technology (CREST), Japan Science and Technology Agency (JST), 7 Gobancho, Kyoto 606-8502, Japan; ifuku.kentaro.2m@kyoto-u.ac.jp; 3 Graduate School for Agriculture, Kyoto University, Kitashirakawa Oiwake-cho, Sakyo-ku, Kyoto 606-8502, Japan

**Keywords:** cyclic electron flow, ferredoxin, NADH dehydrogenase, pgr5, photosynthesis, photosystem I

## Abstract

The molecular entity responsible for catalyzing ferredoxin (Fd)-dependent cyclic electron flow around photosystem I (Fd-CEF) remains unidentified. To reveal the in vivo molecular mechanism of Fd-CEF, evaluating ferredoxin reduction–oxidation kinetics proves to be a reliable indicator of Fd-CEF activity. Recent research has demonstrated that the expression of Fd-CEF activity is contingent upon the oxidation of plastoquinone. Moreover, chloroplast NAD(P)H dehydrogenase does not catalyze Fd-CEF in *Arabidopsis thaliana*. In this study, we analyzed the impact of reduced Fd on Fd-CEF activity by comparing wild-type and pgr5-deficient mutants (*pgr5^hope1^*). PGR5 has been proposed as the mediator of Fd-CEF, and *pgr5^hope1^* exhibited a comparable CO_2_ assimilation rate and the same reduction–oxidation level of PQ as the wild type. However, P700 oxidation was suppressed with highly reduced Fd in *pgr5^hope1^*, unlike in the wild type. As anticipated, the Fd-CEF activity was enhanced in *pgr5^hope1^* compared to the wild type, and its activity further increased with the oxidation of PQ due to the elevated CO_2_ assimilation rate. This in vivo research clearly demonstrates that the expression of Fd-CEF activity requires not only reduced Fd but also oxidized PQ. Importantly, PGR5 was found to not catalyze Fd-CEF, challenging previous assumptions about its role in this process.

## 1. Introduction

In oxygenic photosynthesis, the photon energy absorbed by the light-harvesting systems of both photosystem II (PSII) and photosystem I (PSI) in the photosynthetic electron transport system initiates the excitation of the reaction center chlorophylls—P680 in PSII and P700 in PSI. The excitation of these chlorophylls initiates their catalytic reactions, leading to electron flow from oxidation to reduction: H_2_O oxidation in PSII results in O_2_ evolution, while plastoquinone (PQ) undergoes reduction. Simultaneously, PQ is oxidized through cytochrome (Cyt) *b*_6_/*f* and plastocyanin, eventually leading to the reduction of ferredoxin (Fd) via electron transport carriers, including phylloquinone, Fx, and F_A_/F_B_ in PSI. The electrons from reduced Fd are primarily utilized in the production of NADPH catalyzed by Fd-NADP oxidoreductase. Concomitant with the photosynthetic linear electron flow from H_2_O to NADPH, protons accumulate in the lumen of thylakoid membranes, creating a ΔpH across the thylakoid membranes. These protons originate from water oxidation in PSII and their transport from the stroma to the lumen facilitated by the Q-cycle during PQ oxidation in the Cyt *b*_6_/*f* complex [1,2]. The ΔpH, acting as a proton motive force, drives ATP synthase to generate ATP. These energy compounds—reduced Fd, NADPH, and ATP—produced during the light reaction, play pivotal roles in driving the dark reactions of CO_2_ assimilation and photorespiration in C3 plants.

The photosynthetic linear electron flow faces a potential threat from the generation of reactive oxygen species (ROS). The rate at which NADPH is supplied to ATP, produced in the photosynthetic linear electron flow, surpasses the consumption rate of NADPH to ATP in dark reactions, even under non-photorespiratory conditions [3,4,5,6,7,8,9,10]. This excess supply of NADPH is further heightened under photorespiratory conditions, where a greater amount of ATP is consumed in dark reactions [11,12,13]. Additionally, as CO_2_ assimilation is stimulated by the photosynthetic linear electron flow, the surplus NADPH supply becomes more pronounced. In essence, the photosynthetic linear electron flow, the sole source of electrons for NADPH production in photosynthesis, accumulates NADPH and saturates electrons in the photosynthetic electron transport system. The accumulation of electrons in PSI is evident in the reduction of electron carriers at the acceptor side of PSI, including the Fe/S-series, Fx, F_A_/F_B_, and Fd. This accumulation triggers the reduction of O_2_ to produce O_2_^−^, and the O_2_^−^ subsequently degrades Fe/S compounds, leading to the inactivation of PSI [14,15,16,17,18,19,20,21,22,23].

The issue of a supply rate of NADPH exceeding its demand, as caused by the photosynthetic linear electron flow, finds resolution in the cyclic electron flow around photosystem I (CEF) [3]. Within the CEF, Fd reduced by photosystem I donates electrons to PQ through Fd-quinone oxidoreductase (FQR). In essence, FQR catalyzes Fd-dependent CEF. This cyclic flow induces a ΔpH across thylakoid membranes, generating ATP without producing electrons for NADPH supply. Instead, Fd-CEF promotes the consumption of NADPH and actively contributes to the activation of the dark reaction. In this way, Fd-CEF possesses the potential to alleviate the challenges posed by the photosynthetic linear electron flow.

Until now, the potential threat posed by the photosynthetic linear electron flow has not been thoroughly explored, and the physiological role of the Fd-CEF has not been investigated in terms of oxidative stress. Although CO_2_ assimilation could potentially proceed at an ATP supply-limited rate under photorespiratory conditions, the issue arises when NADPH starts to accumulate. The accumulation of electrons at the acceptor side of PSI, manifesting as the reduction of electron carriers such as Fe/S-clusters, is known to induce ROS production [19]. It is crucial to alleviate the accumulation of NADPH, and this is where the function of Fd-CEF becomes significant. Fd-CEF is proposed to promote the consumption of NADPH by supplying ATP in the dark reaction, thus counteracting the potential ROS production associated with the photosynthetic linear electron flow. This dual role of Fd-CEF in ATP supply and NADPH consumption could play a crucial role in maintaining redox balance and mitigating oxidative stress in the photosynthetic process.

To unravel the physiological function of Fd-CEF, it was imperative to establish an assay system capable of measuring Fd-CEF in vivo. In this study, we monitored the redox reaction of Fd concurrently with chlorophyll fluorescence, P700^+^ and PC^+^ absorbance changes, and net CO_2_ assimilation using intact leaves of *Arabidopsis thaliana*. Successful measurement of the electron flux in Fd-CEF in *Arabidopsis thaliana* had been previously achieved [24]. The oxidation rate of reduced Fd, independent of the photosynthetic linear electron flow—termed the extra oxidation rate of Fd—was designated as the electron flux in Fd-CEF, denoted as vCEF. The regulation of vCEF was found to be linked to the reduction–oxidation state of PQ. Specifically, as PQ became oxidized with an increase in CO_2_ assimilation, vCEF exhibited a corresponding increase [24]. These observed characteristics align with the established model of cyclic electron flow [3]. Importantly, our inability to detect the extra Fd oxidation reaction [24] might be attributed to the suppressed in vivo occurrence of this reaction under conditions of limited photosynthesis. In instances where PQ was highly reduced and the apparent quantum yield of PSII was low, the extra Fd oxidation reaction was inhibited. We posit that the extra Fd oxidation reaction serves as a reflection of Fd-CEF activity.

In this study, we further characterized the impact of the reduction–oxidation state of Fd on Fd-CEF activity in vivo. Fd-CEF necessitates Fd as the electron donor to effectively reduce oxidized PQ [3]. Typically, the redox state of Fd remains constant in response to decreases in both the net CO_2_ assimilation rate and the photosynthetic linear electron flow under constant actinic light intensity [19,24,25,26]. Our recent findings revealed an augmented reduction of Fd in a PGR5-less mutant (*pgr5^hope1^*) of *Arabidopsis thaliana* due to the inhibited oxidation of P700 in PSI [19]. Subsequently, we conducted a comparative analysis of Fd-CEF activity between the wild type (WT) and *pgr5^hope1^* to elucidate the influence of the Fd redox state in vivo. As anticipated, *pgr5^hope1^* exhibited a higher Fd-CEF activity than the WT. This underscores the regulation of Fd-CEF activity by the redox states of both Fd and PQ in vivo. Our study delves into the molecular mechanisms and physiological functions of Fd-CEF in vivo, shedding light on the intricate interplay between Fd redox status and the activity of the cyclic electron flow around PSI.

## 2. Results

**Figure 1:**
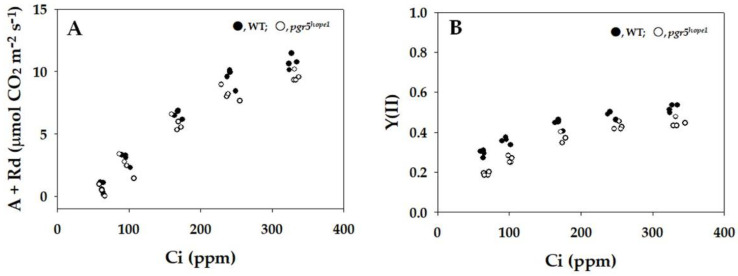
Effects of the intercellular partial pressure of CO_2_ (Ci) on the gross CO_2_ assimilation rate (A + Rd) and apparent quantum yield of photosystem II (PSII) [Y(II)] in wild-type (WT) and *pgr5^hope1^ Arabidopsis*. (**A**) The gross CO_2_ assimilation rates were measured at 400 µmol photons m^−2^ s^−1^ and 21 kPa O_2_, and Y(II) was simultaneously measured. The dark respiration rates (Rd) were measured before starting actinic light illumination. The gross CO_2_ assimilation rates are expressed as A + Rd and were plotted against Ci. (**B**) Y(II) was plotted against Ci. The data were obtained from four independent experiments using leaves attached to four plants of both WT and *pgr5^hope1^* (*n* = 4). The ambient partial pressures of CO_2_ were changed from 400 ppm to 50 through 300, 200, and 100 Pa at 21 kPa O_2_ for the same leaves. Black symbols, WT; white symbols, *pgr5^hope1^*.

**Figure 2:**
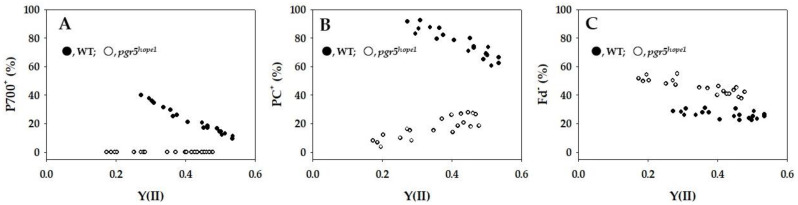
Relationships between P700^+^, PC^+^, Fd^−^, and apparent quantum yield of photosystem II (PSII) [Y(II)]. The data for each parameter were measured in the experiments depicted in Figure 1, simultaneously with the gross CO_2_ assimilation rates and Y(II). (**A**) P700^+^, (**B**) PC^+^, and (**C**) Fd^−^ were plotted against Y(II). The ratios of P700^+^, PC^+^, and Fd^−^ against the total contents are expressed. The data were obtained from four independent experiments using leaves attached to four WT and *pgr5^hope1^* plants (*n* = 4). Black symbols, WT; white symbols, *pgr5^hope1^*.

The impact of intercellular partial pressures of CO_2_ (Ci) on both the gross CO_2_ assimilation rate and the apparent quantum yield of photosystem II [Y(II)] was examined (Figure 1). The gross CO_2_ assimilation rates exhibited a consistent dependence on Ci for both the wild type (WT) and *pgr5^hope1^*. While Y(II) demonstrated a similar Ci dependence in both the WT and *pgr5^hope1^*, the Y(II) values in *pgr5^hope1^* were observed to be lower than those in the WT (Figure 1). The distinctions in Y(II) between WT and *pgr5^hope1^* were particularly evident in the lower range of Y(II), as highlighted in Figure 2.

In Figure 2, the relationships between the parameters P700^+^, PC^+^, and Fd^−^ and Y(II) were depicted. As Y(II) decreased due to lowering Ci, P700 in WT was oxidized, increasing from approximately 10% to 40% (see Figure 2A). In contrast, P700 in *pgr5^hope1^* was not oxidized even as Y(II) decreased (Figure 2A). Similarly, PC in WT was oxidized, ranging from 65% to 90% (Figure 2B). Conversely, the oxidized PC percentage decreased from 20% to 5% with the decrease in Y(II) in *pgr5^hope1^* (Figure 2B). Unlike P700^+^ and PC^+^, Fd^−^ in WT did not show significant changes in response to the decrease in Y(II) (Figure 2C), attributed to the oxidation of P700 in PSI [19]. In contrast, Fd^−^ in *pgr5^hope1^* surpassed that in WT and increased from 40% to 55% with the decrease in Y(II) (Figure 2C). This elevation was attributed to the suppression of P700 oxidation in *pgr5^hope1^*, leading to an enhanced electron flux toward the acceptor side of PS I, ultimately resulting in the reduction of Fd [19].

**Figure 3:**
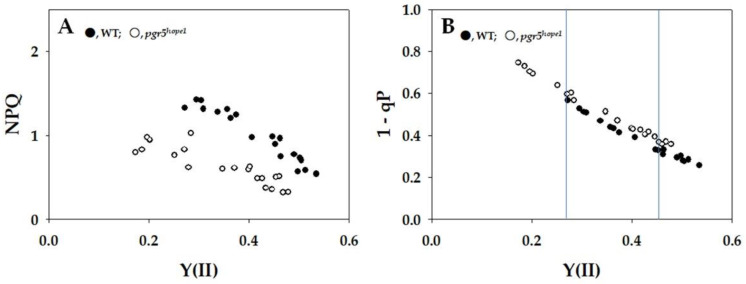
Relationships between non-photochemical quenching (NPQ), plastoquinone reduced state (1 − qP), and apparent quantum yield of photosystem II (PSII) [Y(II)]. The data for each parameter were measured in the experiments depicted in Figure 1 simultaneously with the gross CO_2_ assimilation rates and Y(II). (**A**) NPQ and (**B**) 1 − qP were plotted against Y(II). The two vertical lines were drawn at approximately 0.27 and 0.45 of Y(II), where the values of 1 − qP were the same between WT and *pgr5^hope1^*. These characteristics were used for the comparison of the oxidation of the reduced Fd in Figure 5. The data were obtained from four independent experiments using leaves attached to four WT and *pgr5^hope1^* plants (*n* = 4). Black symbols, WT; white symbols, *pgr5^hope1^*.

In Figure 3, the parameters non-photochemical quenching (NPQ) and plastoquinone reduced state (1 − qP) were plotted against Y(II). An increase in NPQ indicated the enhancement of heat dissipation of photon energy absorbed by PSII. As Y(II) decreased, NPQ in WT rose from approximately 0.5 to 1.5 (Figure 3A). Conversely, NPQ in *pgr5^hope1^* was lower than that in WT, and it increased from 0.2 to 0.9 with the decrease in Y(II) (Figure 3A). The increase in 1 − qP reflects a reduction in the plastoquinone pool. Both WT and *pgr5^hope1^* exhibited the same dependence of 1 − qP on the decrease in Y(II), with 1 − qP rising as Y(II) decreased (Figure 3B). In WT, 1 − qP increased from approximately 0.25 to 0.6 with the decrease in Y(II) (Figure 3B). Similarly, in *pgr5^hope1^*, 1 − qP increased from approximately 0.35 to 0.75 with the decrease in Y(II) (Figure 3B). While the dependence of 1 − qP on Y(II) in *pgr5^hope1^* mirrored that of WT, the values of 1 − qP in *pgr5^hope1^* showed a further increase with lowering Y(II) from 0.25 to 0.15 compared to WT.

**Figure 4:**
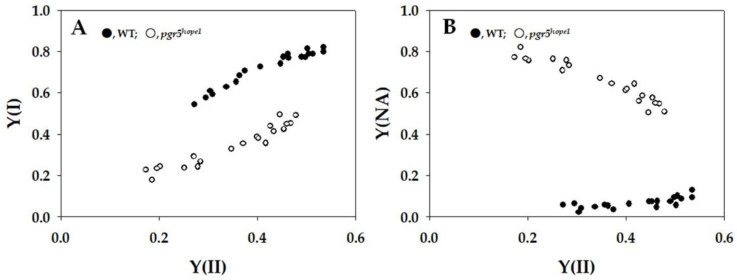
Relationships between the apparent quantum yield of PSI [Y(I)], apparent quantum yield of non-photochemical energy dissipation of photoexcited P700 [Y(NA)], and apparent quantum yield of photosystem II (PSII) [Y(II)]. The data for each parameter were measured in the experiments depicted in Figure 1, simultaneously with the gross CO_2_ assimilation rates and Y(II). (**A**) Y(I) and (**B**) Y(NA) were plotted against Y(II). The data were obtained from four independent experiments using leaves attached to four WT and *pgr5^hope1^* plants (*n* = 4). Black symbols, WT; white symbols, *pgr5^hope1^*.

In Figure 4, the parameters related to photosystem I (PSI), namely Y(I) and Y(NA), were shown. As Y(II) decreased, Y(I) in WT declined from approximately 0.8 to 0.5 (Figure 4A). Conversely, in *pgr5^hope1^*, Y(I) decreased from about 0.5 to 0.2 with the decrease in Y(II) (Figure 4A). Notably, the dependence of Y(I) on Y(II) in *pgr5^hope1^* differed from that in WT, and the values of Y(I) in *pgr5^hope1^* were consistently lower than those in WT. Turning to Y(NA), in WT, it maintained lower values ranging from 0.1 to 0.05 as Y(II) decreased (Figure 4B). In contrast, Y(NA) in *pgr5^hope1^* was higher than in WT. Specifically, in *pgr5^hope1^*, Y(NA) increased from 0.5 to 0.8 as Y(II) decreased (Figure 4B). This suggests an acceleration of the limitation of the oxidation of the excited P700 in the photo-oxidation cycle of P700 in PSI (Figure 2C). Furthermore, in *pgr5^hope1^*, Y(NA) appeared to be related to the dependence of Fd reduction on Y(II).

To delve deeper into this relationship, a statistical comparison was conducted between the dependence of Y(NA) on the reduced state of Fd in WT and *pgr5^hope1^* (Supplemental Figure S5). ANCOVA analysis revealed a significant interaction between plants (WT and *pgr5^hope1^*) and the reduced Fd (*p* < 0.01). Subsequently, correlation analysis between Y(NA) and the reduced Fd was performed for each plant. In WT, ANOVA of the regression analysis showed no significant relationship between Y(NA) and the reduced Fd. However, in *pgr5^hope1^*, ANOVA of the regression analysis demonstrated a significant relationship between Y(NA) and the reduced Fd (F value 35.29, *p* < 0.01). The regression line was Y(NA) = −0.0717 + 0.01589** × Fd^-^ (** *p* < 0.01), indicating a significant slope. Thus, the correlation between Y(NA) and the reduced Fd was observed exclusively in *pgr5^hope1^*.

**Figure 5:**
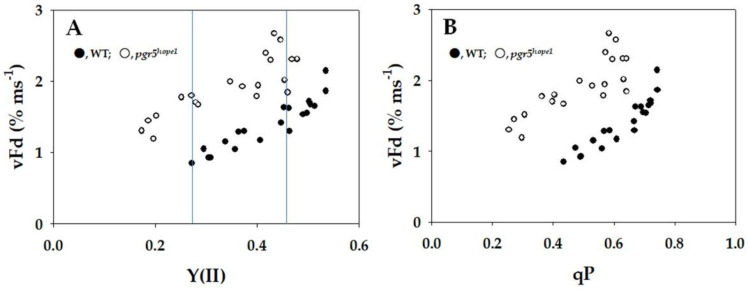
Relationships between the apparent quantum yield of photosystem II (PSII) [Y(II)], plastoquinone oxidized state (qP), and vFd. The data for each parameter were measured in the experiments depicted in Figure 1 simultaneously with the gross CO_2_ assimilation rates and Y(II). (**A**) Y(II) was plotted against vFd. (**B**) qP was plotted against vFd. In the experiments shown in Figure 1, the oxidation rate of Fd was determined by DIRK analysis (see “Section 4”). To determine the oxidation rate of Fd^−^ (vFd) under illuminated conditions, actinic light was transiently turned off for 400 ms. The initial slope of the decrease in Fd^−^ indicates vFd. These data were obtained at a steady state, which was confirmed by the achievement of stable conditions for both gross CO_2_ assimilation and Y(II). The two vertical lines were drawn at approximately 0.27 and 0.45 of Y(II) to compare vFd between WT and *pgr5^hope1^*. The data were obtained from four independent experiments using leaves attached to four WT and *pgr5^hope1^* plants (*n* = 4). Black symbols, WT; white symbols, *pgr5^hope1^*.

Figure 5A illustrated the relationship between the oxidation rate of reduced Fd (vFd) and Y(II) in both WT and *pgr5^hope1^*. In WT, vFd was proportional to Y(II) in the range of Y(II) below 0.4. Beyond 0.4 of Y(II), Y(II) became saturated against vFd, indicating the discovery of excess turnover of Fd, indicative of Fd-CEF activity. The behavior of vFd against Y(II) in *pgr5^hope1^* was similar to that in WT (Figure 5A), but the values of vFd in *pgr5^hope1^* were higher in the range of Y(II) compared to WT. These findings suggest that Fd-CEF was activated in *pgr5^hope1^*. Supplemental Figure S1A provided a comparison of the typical kinetics of the oxidation of Fd after turning off the actinic light in the dark-interval relaxation kinetics (DIRK) analysis between WT and *pgr5^hope1^* at approximately the same two values of Y(II). At approximately 0.45 of Y(II), the initial decay rate of the reduced Fd in *pgr5^hope1^* was larger than that in WT, indicating a higher Fd-CEF activity. The reduced level of Fd before turning off the actinic light showed a reduced level at the steady state. At approximately 0.27 of Y(II) at lower Ci, the initial decay rate of the reduced Fd in *pgr5^hope1^* was also larger than that in WT (Supplemental Figure S1B). Additionally, Figure 5B demonstrates that vFd exhibited a dependence on the increase in qP. The increase in qP, reflecting the oxidation of PQ, stimulated the oxidation rate of Fd, resulting in excessive vFd, observed with the increase in Y(II) in both WT and *pgr5^hope1^*. These observations suggest that Fd-CEF activity is induced by the oxidation of PQ, which is a consequence of the enhanced photosynthetic linear electron flow. Furthermore, the vFd in *pgr5^hope1^* was also larger than in WT, indicating that the higher reduced state of Fd stimulates Fd-CEF activity in vivo.

## 3. Discussion

In this present study, we employed *pgr5^hope1^* as our experimental model due to its manifestation of a higher reduced state of Fd at the steady state. Consequently, we anticipated that *pgr5^hope1^* would serve as a suitable material for investigating the impact of Fd on Fd-CEF activity in vivo. We conducted a comparative analysis of Fd-CEF activity in *Arabidopsis thaliana* wild type (WT) and *pgr5^hope1^*. The *pgr5^hope1^* mutant exhibited comparable values for both the gross CO_2_ assimilation rate and the reduction–oxidation level of PQ when compared to WT (see Figure 1) [19,27]. The oxidation rate of the reduced Fd displayed a nonlinear relationship with Y(II) in both WT and *pgr5^hope1^* (Figure 5A). The increase in vFd deviated from the rise in Y(II) in both cases, indicating the presence of excessive vFd unrelated to photosynthetic linear electron flow—the electron flux in Fd-CEF (Figure 5A) [24]. Furthermore, we observed a deviation in the relationship between vFd and qP in both WT and *pgr5^hope1^* (Figure 5B). The increase in qP reflected the oxidation of the reduced PQ induced by the stimulation of photosynthetic linear electron flow. These findings align with a previous report [24], suggesting that the expression of excessive vFd corresponds to a characteristic feature of Fd-CEF. In essence, the appearance of Fd-CEF required PQ oxidation in both WT and *pgr5^hope1^*, aligning with the molecular mechanism of Fd-CEF (Supplemental Figure S2) [3].

Moreover, we observed an elevated oxidation rate of reduced Fd in *pgr5^hope1^* compared to WT, as illustrated in Figure 5A. This implies that the electron flux of Fd-CEF in *pgr5^hope1^* was enhanced. In the Fd-CEF process, the reduced Fd serves as the electron donor, transferring electrons to PQ through FQR. The electron acceptor in this process is the oxidized PQ. The electron flux in Fd-CEF (vCEF) is directly proportional to the product of the concentrations of both the reduced Fd ([Fd^-^]) and the oxidized PQ ([PQ]). Additionally, it depends on the activity of FQR and the rate constant (k), as expressed by the equation:

vCEF = k × [PQ] × [Fd^-^] (1)

If PQ reached complete reduction, the electron flux of Fd-CEF became zero, even if Fd was in a reduced state (Supplemental Figure S2) [3,24]. Conversely, when PQ was entirely oxidized, the activity of Fd-CEF was also zero, since Fd lacked electrons for the reduction of PQ [3]. Similarly, if Fd was fully reduced with PQ also in a completely reduced state, Fd-CEF could not function. Furthermore, if Fd was entirely oxidized with PQ completely oxidized, Fd-CEF would not be operational. In our current investigation, the reduction level of Fd in *pgr5^hope1^* surpassed that in WT, and the further reduction of Fd was facilitated by the decline in photosynthetic linear electron transport (Figure 2C). This reduction was a consequence of the suppressed P700 oxidation in *pgr5^hope1^* (Figure 2A). Unlike WT, where the rate-determining step in the P700 photo-oxidation reduction cycle is the oxidation of the excited P700 by the electron acceptor in PSI, *pgr5^hope1^* demonstrated a distinct pattern. In *pgr5^hope1^*, this rate-determining step was observed as a larger Y(NA) and a higher reduced level of Fd (Figure 2 and Figure 4). This shift was attributed to the lower ΔpH across thylakoid membranes in *pgr5^hope1^* compared to WT (Supplemental Figure S3). The diminished ΔpH in *pgr5^hope1^* resulted from a higher value of H^+^-conductance (gH^+^) compared to WT [28]. However, the mechanism by which gH^+^ is decreased in *pgr5^hope1^* has not been elucidated. Notably, the observed lower ΔpH in *pgr5^hope1^* did not impede the oxidation of the reduced PQ by the cytochrome *b*_6_/*f*-complex. Consequently, the rate-determining step in the P700 photo-oxidation reduction cycle in *pgr5^hope1^* shifted from the reduction of the oxidized P700 to the oxidation of the excited P700. This explains the intensified reduction in Fd in *pgr5^hope1^*, particularly in response to the suppression of photosynthetic linear electron transport. According to the Fd-CEF activity expression model (Equation (1)), the increase in [Fd^-^] results in an elevation of vCEF. Indeed, at identical qP values (e.g., 0.4 and 0.6), indicating the same [PQ], vFd in *pgr5^hope1^* exceeded that in WT (Figure 5B). These observations align with the behaviors predicted by the Fd-CEF model (Supplemental Figure S2) [3]. In essence, Fd-CEF necessitates the presence of both oxidized PQ and reduced Fd in vivo.

The role of Fd-CEF in inducing ΔpH across thylakoid membranes has been previously explored [1,2,3,7,8,29,30,31,32,33,34,35,36,37,38,39,40,41,42]. The dependencies of vFd on both Y(II) and qP, as illustrated in Figure 5, indicated that the acceleration of Fd-CEF was concurrent with an increase in CO_2_ assimilation in response to elevated Ci levels. However, despite the rise in Fd-CEF activity, ΔpH across the thylakoid membranes remains constant (Supplemental Figure S3). This implies that the ΔpH induced by Fd-CEF is dissipated by the increased CO_2_ assimilation, where the usage of ATP is augmented. Unless Fd-CEF is stimulated by an increase in Ci, the ΔpH would not be maintained by the heightened CO_2_ assimilation at higher Ci levels. The dependencies of ΔpH on the increase in Ci were consistent between WT and *pgr5^hope1^* (Supplemental Figure S3). However, the ΔpH across thylakoid membranes in *pgr5^hope1^* was lower than in WT, in line with previous reports [28,42]. The reduced ΔpH in *pgr5^hope1^* resulted from a larger gH^+^ compared to WT (Supplemental Figure S3). The molecular mechanism underlying this increased gH^+^ in *pgr5^hope1^* remains unclear. Despite the lower ΔpH in *pgr5^hope1^*, the CO_2_ assimilation rates were almost identical to WT (Figure 1). This suggests that the diminished ΔpH in *pgr5^hope1^* was sufficient to drive CO_2_ assimilation, a phenomenon supported by the stimulated Fd-CEF activity (Figure 5). Without the acceleration of Fd-CEF in *pgr5^hope1^*, the ΔpH could not be sustained, jeopardizing the functionality of CO_2_ assimilation.

In *pgr5^hope1^*, non-photochemical quenching (NPQ) was observed to be lower compared to WT, as depicted in Figure 3. The induction of NPQ requires acidification of the luminal side of thylakoid membranes [33,43]. Consequently, the lower ΔpH in *pgr5^hope1^* may explain the inability to induce higher NPQ. On the contrary, the behavior of NPQ in WT in response to both the increase in the gross CO_2_ assimilation rate and the photosynthetic linear electron flow rate mirrored that of *pgr5^hope1^* (Figure 3). NPQ decreased with the rise in both the gross CO_2_ assimilation rate and the photosynthetic linear electron flow rate, despite ΔpH remaining unchanged in both WT and *pgr5^hope1^*, as described earlier. NPQ is also influenced by the reduction–oxidation state of PQ and Y(II) [44,45]. Consequently, NPQ decreased with increases in both qP and Y(II).

In this study, we have further substantiated the expression model of Fd-CEF activity proposed in Supplemental Figure S2 [3]. The *pgr5^hope1^* mutant exhibited a higher reduction in Fd compared to WT (Figure 2). As anticipated, this led to an enhancement in the electron flux of Fd-CEF in *pgr5^hope1^*, as depicted in Figure 5. Both pgr5 and NDH have been recognized as potential mediators of Fd-CEF [6,7]. Mutants of these components displayed a suppression of the increase in the minimum yield of Chl fluorescence (Fo) after actinic light illumination was turned off in vivo [7,35]. Additionally, the reduced Fd-dependent increase in Fo was inhibited in the isolated thylakoid membranes from both pgr5- and NDH-less *Arabidopsis thaliana* [6]. The increase in Fo was attributed to the reduction of PQ by the reduced Fd, and thus, the Fd-dependent increase in Fo has been utilized as a measure of FQR activity [6]. However, it is noteworthy that the reduced Fd increased Fo even in the presence of the inhibitor (DCMU) of the electron transport in PSII [46]. Furthermore, Fd was observed to reduce cytochrome *b*_559_ in PSII, and this reduction was inhibited by antimycin A [47]. These observations imply that both pgr5 and NDH contribute to the reduction of PQ through PSII. Although the Fd-dependent reduction of PSII catalyzed by pgr5 and NDH can form the electron flow pathway in CEF around PSI, it is considered to be relatively small compared to the electron flux in photosynthetic linear electron flow. This conclusion is supported by the fact that the electron flux in PSII (Jf) estimated by Chl fluorescence, Y(II), exhibits a positive linear relationship with the electron fluxes (Jg) into both net CO_2_ assimilation and photorespiration [48,49,50,51]. This suggests that no additional electron flux beyond photosynthetic linear electron flow is detected. Therefore, the observed electron flux in the Fd redox reaction, not associated with photosynthetic linear electron flow, constitutes the cyclic electron flow from PSI to PQ through the electron transport carrier localized between PSII and PSI, revealing the Fd-dependent cyclic electron flow around PSI, Fd-CEF. Our current and previous findings unequivocally demonstrate that Fd-CEF is driven by a new mediator [24]. The most compelling candidate for this mediator, FQR, is Cyt *b*_6_/*f*-complex [52,53,54]. The Cyt *b*_6_/*f*-complex possesses a potential binding site for Fd, situated close to the heme *c* location, composed of basic amino groups. The acidic region of Fd would bind to the Cyt *b*_6_/*f*-complex at this site. The reduced heme *c* would then transfer electrons to the low-potential heme *b* in the Cyt *b* subunit of the Cyt *b*_6_/*f*-complex [52,54]. Subsequently, the reduced heme *b* donates electrons to the oxidized PQ and/or the one-electron reduced PQ in the Q-cycle of the Cyt *b*_6_/*f*-complex. This cyclic electron flow accelerates the Q-cycle and contributes to ΔpH formation. Further research is required to conclusively identify the mediator for Fd-CEF.

Subsequently, we have recognized what we consider to be the most critical insight into the physiological function of Fd-CEF in *pgr5^hope1^*. The *pgr5^hope1^* mutant demonstrates elevated H^+^-conductance, as reported by various researchers [42,55], and this is evident in Supplemental Figures S3 and S4. Without Fd-CEF inducing acidification of the luminal space of thylakoid membranes, the proton motive force required for ATP production should not be sustained. The heightened electron flux in Fd-CEF of *pgr5^hope1^* appears to play a compensatory role, counteracting the rapid loss of proton motive force by facilitating ΔpH formation across thylakoid membranes. In other words, as illustrated in Figure 1 and Figure 5, the increased activity of Fd-CEF in *pgr5^hope1^* effectively drives CO_2_ assimilation at a rate comparable to that of WT.

## 4. Materials and Methods

### 4.1. Plant Materials and Growth Conditions

*Arabidopsis* plants (*Arabidopsis thaliana* WT and *pgr5^hope1^*) were cultivated under the same condition as that reported by the previous study [24]. These plants of both WT and *pgr5^hope1^* were analyzed with crr4, which was deficient mutant in chloroplastic NADH dehydrogenase [24]. The comparative analysis between WT and crr4 was already reported [24]. In the present research, *pgr5^hope1^* were comparatively analyzed with WT.

### 4.2. Contents of Both Chlorophyll and Nitrogen

The contents of both chlorophyll and nitrogen in the leaves of *Arabidopsis* plants were determined by the method reported in the previous study [24].

### 4.3. Simultaneous Measurements of Chlorophyll Fluorescence, P700, Plastocyanin, and Ferredoxin with CO_2_/H_2_O-Exchange

One set of *Arabidopsis* plants (*Arabidopsis thaliana* WT and *pgr5^hope1^*) grown under the above growth conditions were used for the simultaneous analysis of Chlorophyll fluorescence, P700, Plastocyanin, and Ferredoxin with CO_2_/H_2_O-exchange reported by the previous study [24]. The other set different from the above set were used for the simultaneous analysis of the electrochromic shift (ECS) signal with CO_2_/H_2_O-exchange, reported by the previous study [56].

The total photoreducible ferredoxin (Fd) signal originated from Fe/S signal [57]. The ratio of Fd to P700 in PSI was approximately 5 [58,59]. Furthermore, the leaves of tobacco plants had approximately 5 μmol Fd m^−2^ leaf area [41] and approximately 1 μmol P700 m^−2^ leaf area [60]. That is, the ratio of Fd to P700 in PSI was much closer to that of spinach leaves [58]. Then, we hypothesized that the amount of Fd in *Arabidopsis thaliana* was close to these values. The PSI complex contains Fx and F_A_/F_B_, in which Fe/S-clusters are the electron transfer carriers. That is, the Fe/S signal as Fd occupied less than 60% of the total Fe/S signal. Furthermore, the electron flux from Fx to NADP^+^ through Fd is limited by the oxidation of the reduced Fd [61]. If the observed Fe/S signal was lower than 60%, we monitored the redox reaction of Fd.

### 4.4. Simultaneous Measurements of Electrochromic Shift Signal with CO_2_/H_2_O-Exchange

Electrochromic shift (ECS) signal with CO_2_/H_2_O-exchange in *Arabidopsis* plants (*Arabidopsis thaliana* WT and *pgr5^hope1^*) were simultaneously analyzed by the method reported in the previous study [56]. The magnitude of the ECS signal was analyzed by DIRK analysis [62,63,64] and normalized as follows [65]. A single turnover flash (10 μs) was used to illuminate the leaf under far-red light. Then, the single turnover flash induced PSII-dependent production of ECS signal, which corresponds to the membrane potential induced by single-charge separation of P680 in PSII. The average value of a single turnover (ST) flash-induced ECS signal (ECS_ST_) was (4.09 ± 0.07) × 10^−3^ ΔI/Io (*n* = 4) (WT) and (4.0 ± 0.4) × 10^−3^ ΔI/Io (*n* = 4) (*pgr5^hope1^*). Then, the measured ECS signal was divided by ECS_ST_ and was used as the normalized ECS signal (ECS_N_) [56] (Equation (2)):

ECS_N_ = ECS/ECS_ST_ (2)

The contribution of both ΔpH and Δψ to the total ECS signal was separately evaluated after turning off the AL illumination over longer periods of darkness [63]. The relative H^+^ consumption rate vH^+^ is the decay rate of ECS signal, which was evaluated by DIRK analysis [63]. The half-time of the decay reflects the H^+^ conductance (gH^+^) [63]. The vH^+^ is proportional to both ECS_N_ and gH^+^, as described by (Equation (3)):

vH^+^ = gH^+^ × ECS_N_ (3)

### 4.5. Statistical Analytics

Statistical analysis of the corresponding data in both the text (ANCOVA, ANOVA, regression analysis) and Supplemental Table S1 (CI, confidential interval) was performed using the commercial software JMP8 (ver. 14.2.0, SAS Institute Inc., Cary, NC, USA).

## Data Availability

Data are contained within the article and Supplemental Materials.

## Supplementary Materials

The following supporting information can be downloaded at https://www.mdpi.com/article/10.3390/ijms25052677/s1.
