# Supplementary figures and images for: Enhanced Reduction of Ferredoxin in PGR5-Deficient Mutant of Arabidopsis thaliana Stimulated Ferredoxin-Dependent Cyclic Electron Flow around Photosystem I
Source: Int J Mol Sci. 2024 Feb 26;25(5):2677. doi: 10.3390/ijms25052677 (PMC10931702; doi:10.3390/ijms25052677)

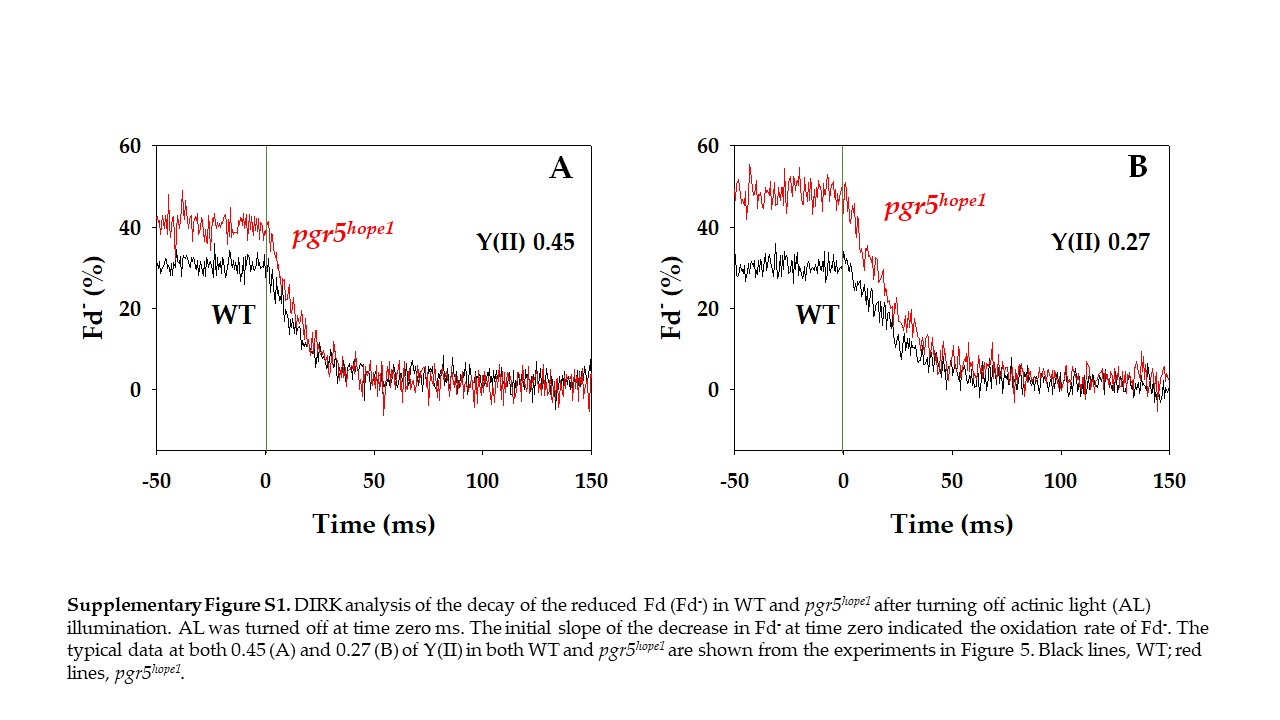

Supplement: Supplementary file 1 [file ijms-25-02677-s001.zip › SupplementalFigure_1.JPG]

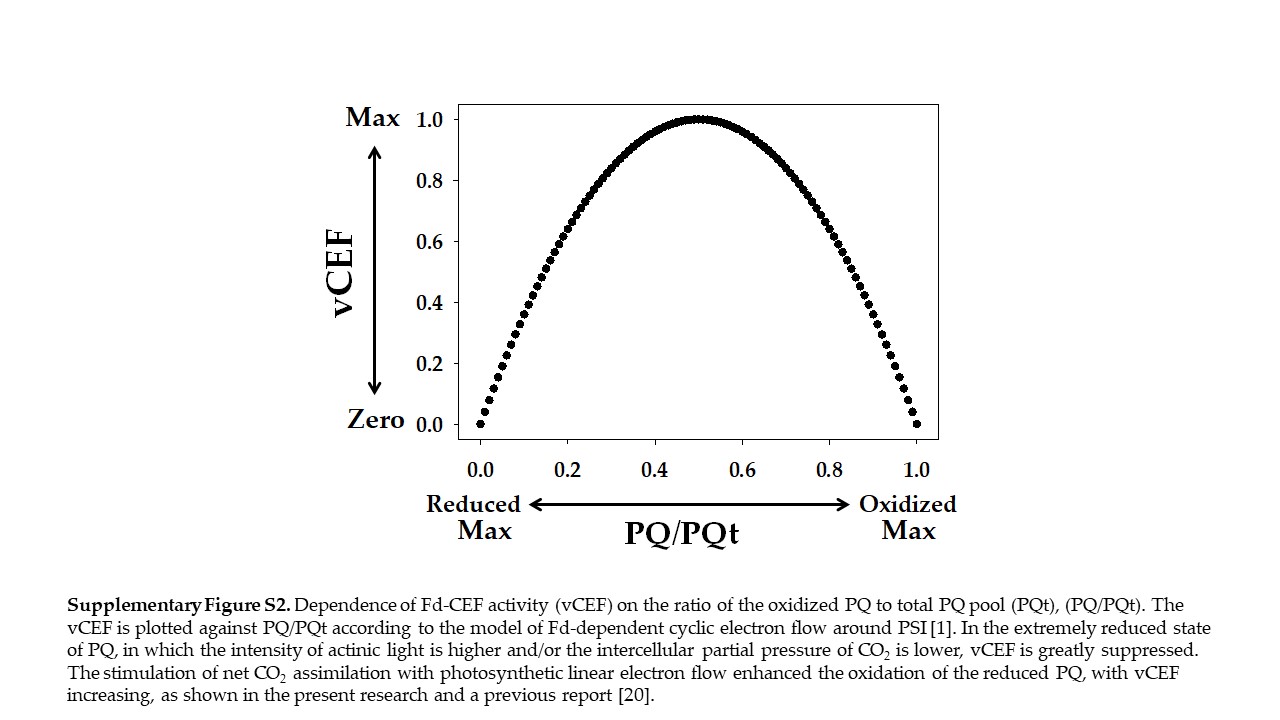

Supplement: Supplementary file 1 [file ijms-25-02677-s001.zip › SupplementalFigure_2.JPG]

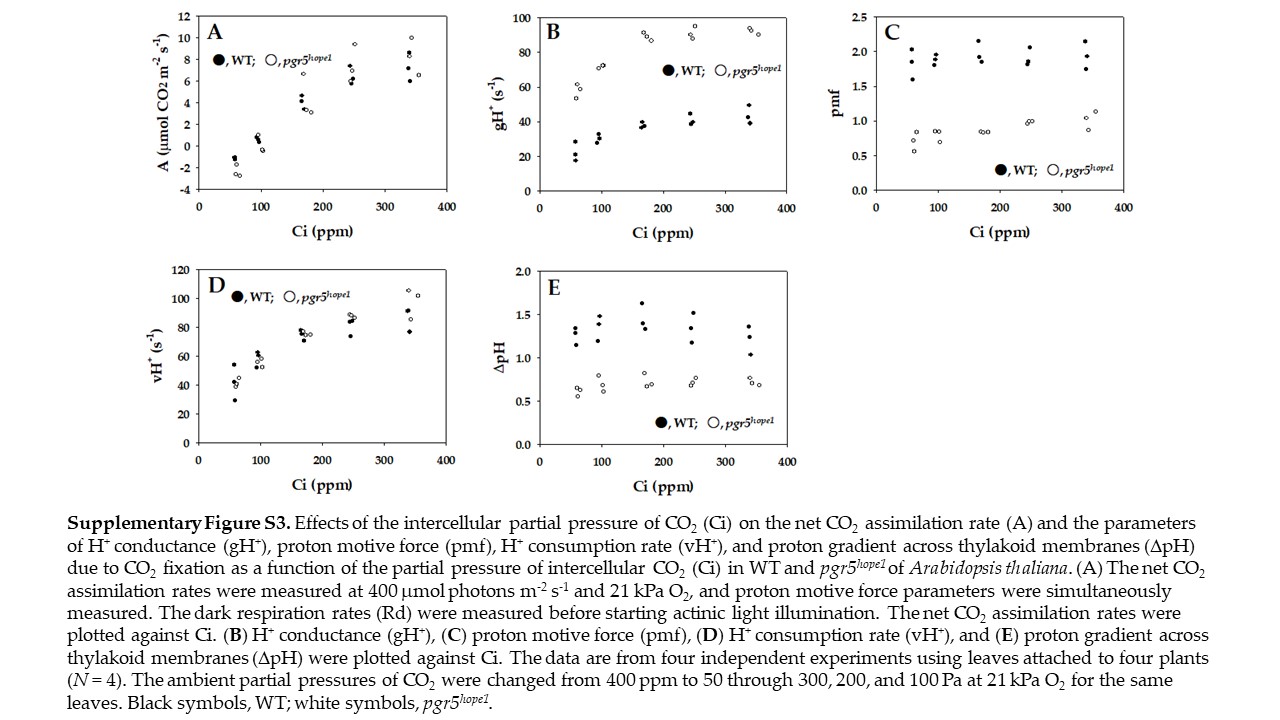

Supplement: Supplementary file 1 [file ijms-25-02677-s001.zip › SupplementalFigure_3.JPG]

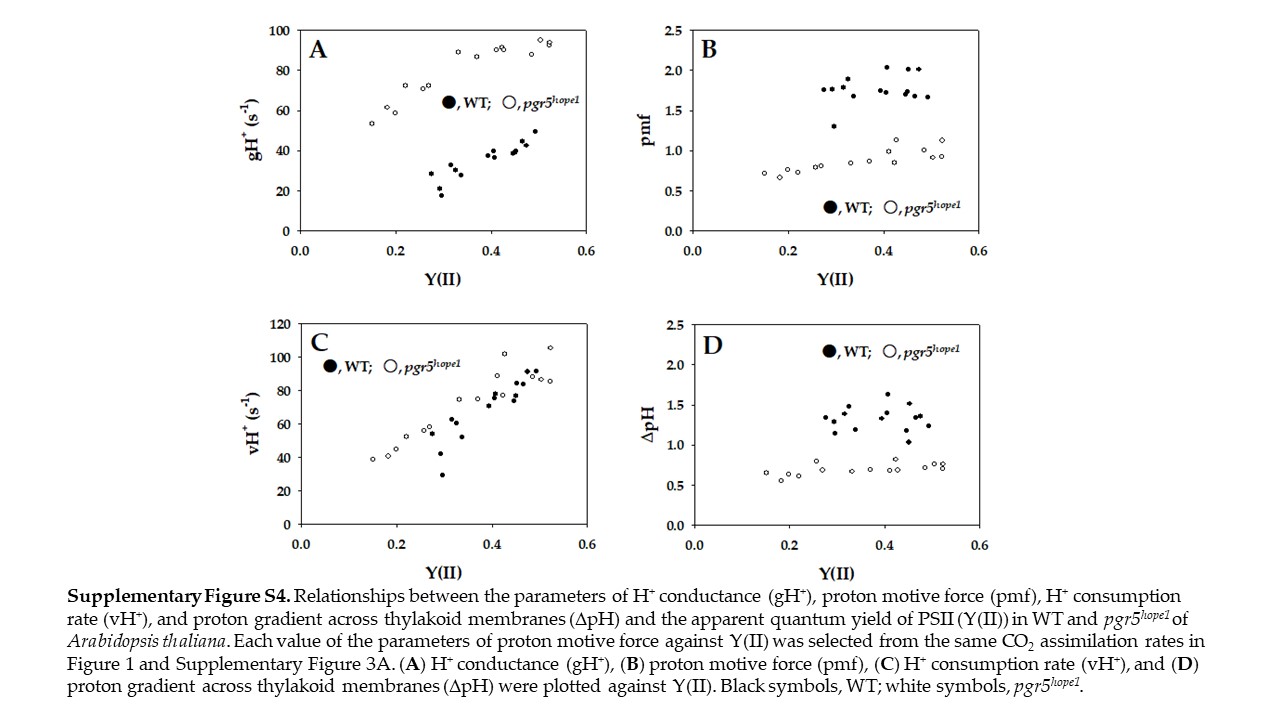

Supplement: Supplementary file 1 [file ijms-25-02677-s001.zip › SupplementalFigure_4.JPG]

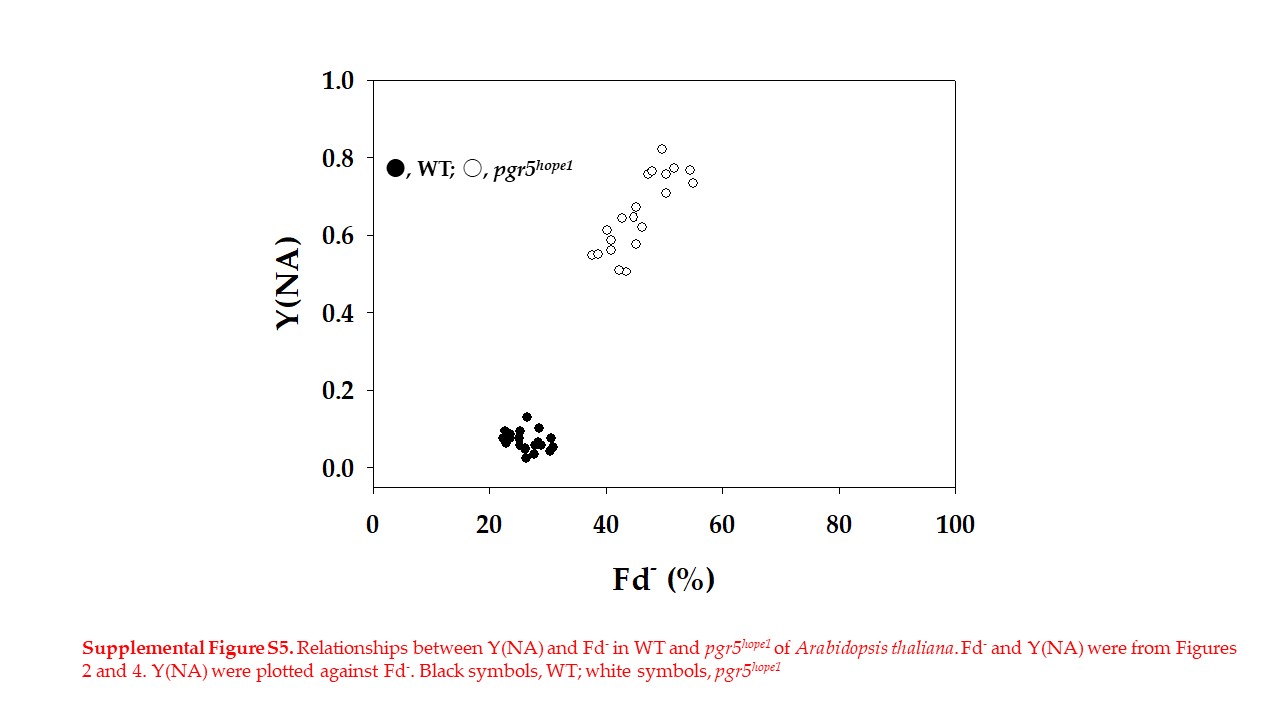

Supplement: Supplementary file 1 [file ijms-25-02677-s001.zip › SupplementalFigure_5.JPG]

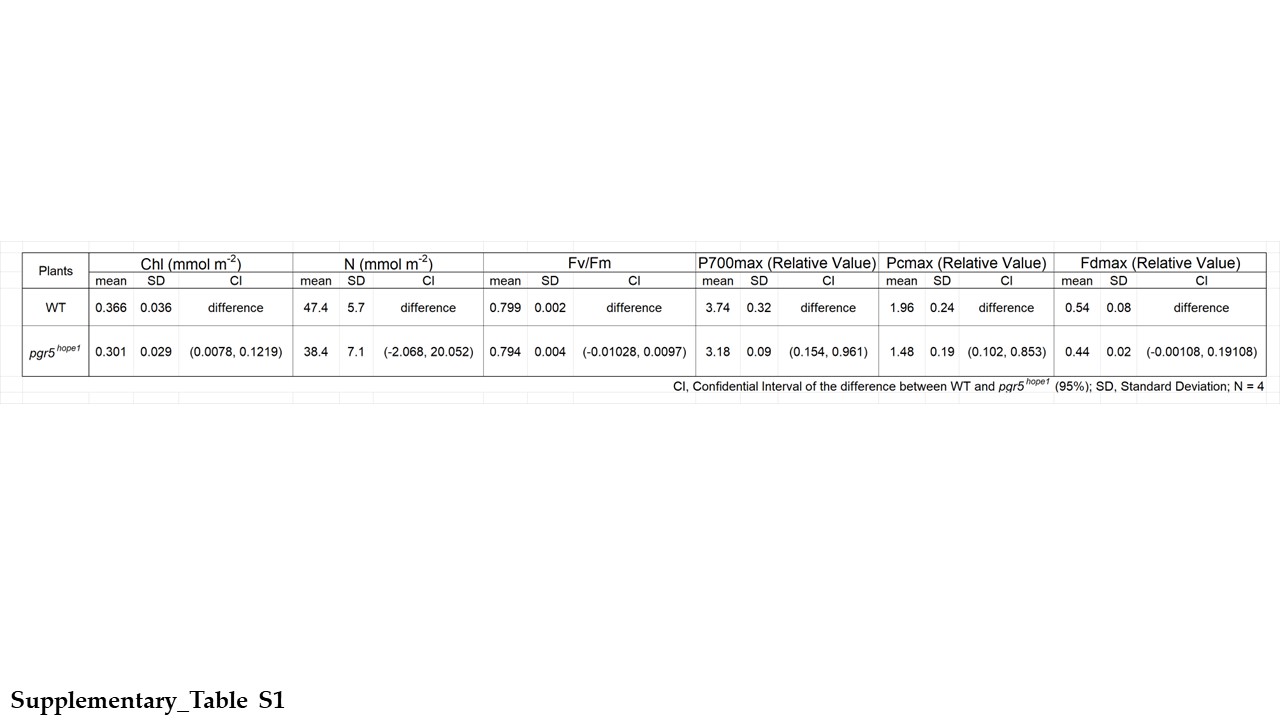

Supplement: Supplementary file 1 [file ijms-25-02677-s001.zip › Supplemental_Table1.JPG]
